# Impact of Continuous Positive Airway Pressure Therapy on Diabetes Control in Patients With Obstructive Sleep Apnea at a Tertiary Hospital in Saudi Arabia: A Retrospective Study

**DOI:** 10.7759/cureus.99756

**Published:** 2025-12-21

**Authors:** Abdulelah A Alzahrani, Ahmed A Alzahrani, Abdulrahman M Alanazi, Shaheen M Almsaeed, Saleh A Alghamdi, Abir Said

**Affiliations:** 1 Internal Medicine, King Fahd University Hospital, Imam Abdulrahman Bin Faisal University Hospital, Al-Khobar, SAU; 2 Internal Medicine (Pulmonolgy), King Fahd University Hospital, Imam Abdulrahman Bin Faisal University Hospital, Al-Khobar, SAU

**Keywords:** continuous positive airway pressure, diabetes, glycated hemoglobin, glycemic control, obstructive sleep apnea

## Abstract

Background: Obstructive sleep apnea (OSA) is highly prevalent among patients with type 2 diabetes mellitus (T2DM) and is recognized as an independent contributor to insulin resistance and poor glycemic control through mechanisms such as intermittent hypoxia, sleep fragmentation, and sympathetic overactivity. Continuous positive airway pressure (CPAP) therapy is the standard treatment for OSA and has been shown to improve respiratory parameters; however, evidence regarding its metabolic benefits remains inconsistent, particularly in real-world clinical practice where adherence and disease severity vary.

Objective: To evaluate changes in glycemic and metabolic parameters following CPAP therapy in patients with coexisting OSA and T2DM and to examine the influence of CPAP adherence, OSA severity, and baseline glycemic control on treatment outcomes.

Methods: A retrospective cohort study was conducted at King Fahad Hospital of the University, including adults diagnosed with both OSA and T2DM between 2018 and 2025. Demographic data, polysomnography results, and metabolic parameters were obtained from electronic medical records. Glycemic and metabolic markers were compared before and after six months of CPAP therapy. Subgroup analyses were performed according to CPAP adherence status, apnea severity, and baseline glycated hemoglobin (HbA1c) levels. Statistical analyses were performed using SPSS version 26 (IBM Corp., Armonk, NY, USA).

Results: A total of 112 patients were included in the study with a mean age of 54.2 years, and 57.1% were male. After six months of CPAP therapy, median HbA1c levels significantly decreased from 7.15% to 6.9%. Greater reductions in HbA1c were observed among patients adherent to CPAP therapy (7.55% to 6.25%), those with severe OSA (7.1% to 6.8%), and patients with baseline HbA1c levels greater than 9% (10% to 8.6%). No statistically significant changes were observed in lipid profile parameters or blood pressure measurements.

Conclusion: CPAP therapy was associated with improved glycemic control in patients with OSA and T2DM, particularly among those with higher adherence to CPAP, severe OSA, and poor baseline glycemic control.

## Introduction

Obstructive sleep apnea (OSA) is a prevalent sleep disorder characterized by recurrent episodes of complete or partial upper airway collapse during sleep, leading to oxygen desaturation, sleep fragmentation, and frequent arousals [1]. OSA affects nearly one billion adults worldwide, with its prevalence increasing due to rising obesity rates and an aging population [2,3]. Obesity, the primary risk factor for OSA, affects over 890 million adults worldwide according to the World Health Organization [3,4]. Other well-established risk factors include central fat distribution, advanced age, male sex, and anatomical abnormalities of the upper airway and facial structure. Additionally, OSA is strongly associated with metabolic disorders such as type 2 diabetes mellitus (T2DM), metabolic syndrome, acromegaly, and hypothyroidism [1].

OSA severity in adults is classified based on the number of apnea and hypopnea events per hour, measured using the apnea-hypopnea index (AHI). According to this classification, mild OSA is defined as five to 15 events per hour, moderate OSA as more than 15 to 30 events per hour, and severe OSA as more than 30 events per hour [1,5].

OSA can result in severe and potentially life-threatening complications, including hypertension, myocardial infarction, cerebrovascular accidents, depression, and sleep-related accidents [6-10]. Notably, OSA and T2DM share common risk factors, particularly obesity and aging, contributing to their frequent coexistence [11]. Furthermore, there is a bidirectional relationship between these two conditions; OSA disrupts sleep architecture, leading to hormonal dysregulation and insulin resistance, which increases the risk of developing T2DM. Conversely, T2DM may contribute to OSA through mechanisms such as peripheral neuropathy and alterations in central respiratory control, though this relationship is not yet fully understood [12,13].

Continuous positive airway pressure (CPAP) therapy is the gold standard treatment for OSA, maintaining airway patency and preventing apnea and hypopnea episodes [14]. Although evidence exists regarding its impact on glycemic control in patients with T2DM, some inconsistencies in the findings persist. Some studies suggest that CPAP therapy may improve insulin sensitivity and glycemic markers, while others report no significant effects [15-18]. Additionally, research on the influence of CPAP on other chronic conditions, such as hypertension and dyslipidemia, is still emerging. A major limitation in existing studies is the relatively small sample sizes, which restricts the generalizability of findings and prevents definitive conclusions. From a pathophysiological perspective, CPAP therapy may improve glycemic control by reversing intermittent hypoxia and sleep fragmentation, both of which are known to promote insulin resistance through sympathetic nervous system activation, hormonal dysregulation, and systemic inflammation [19]. Clinical evidence suggests that glycemic improvement with CPAP therapy is more likely to occur with adequate adherence and longer treatment duration. Randomized clinical trials and meta-analyses have demonstrated modest but significant reductions in HbA1c following sustained CPAP use, particularly in patients with poor baseline glycemic control, supporting a biologically plausible and evidence-based link between CPAP therapy and glucose metabolism [16,17].

Given the increasing burden of both OSA and T2DM, further investigation is needed to clarify the effects of CPAP therapy on glycemic control and metabolic outcomes. The primary objective of this study was to determine whether CPAP therapy leads to significant improvement in glycemic control, assessed by changes in HbA1c after six months of therapy. The secondary objectives were to examine the associations between glycemic improvement and CPAP adherence, OSA severity, and baseline HbA1c levels. By addressing these objectives, we aim to clarify the clinical role of CPAP therapy in improving metabolic outcomes among patients with coexisting OSA and type 2 diabetes mellitus. By including a larger patient population and addressing the gap in retrospective studies, we aim to provide more robust and clinically relevant insights that can optimize management strategies and improve health outcomes for patients suffering from both conditions.

## Materials and methods

Study design and setting

A retrospective cohort, single-center study was conducted between November 2018 and January 2025 at King Fahad Hospital of the University (KFHU), Imam Abdulrahman Bin Faisal University (IAU), Al-Khobar, Eastern Province, Saudi Arabia, a tertiary referral hospital. The study involved a comprehensive review of electronic health records (EHRs) to assess the impact of CPAP therapy on glycemic control in patients with T2DM and OSA. The study protocol was approved by the Institutional Review Board (IRB-UGS-2024-01-745) of KFHU, and the study was conducted in accordance with the Declaration of Helsinki. All patient data were anonymized and de-identified prior to analysis to maintain confidentiality and privacy.

Study population

The study included adult patients aged between 18 and 80 years with confirmed diagnoses of both T2DM and OSA, as determined by polysomnography (PSG) with an apnea-hypopnea index (AHI) greater than five events per hour accompanied by symptoms of OSA, or an AHI greater than 15 regardless of the symptoms. T2DM was defined as having a fasting plasma glucose of 126 mg/dL or more, and HbA1c of ≥6.5. Eligible patients were required to have been on CPAP therapy for more than six months during the study period and have complete medical records. Exclusion criteria included patients with respiratory failure, severe cardiovascular disease (ejection fraction <35%), end-stage renal disease, cancer, or those who had undergone bariatric surgery. Additionally, patients with newly diagnosed diabetes (within one year of diagnosis) or incomplete medical records were excluded. Out of 778 patient files reviewed, a total of 112 patients met the inclusion criteria and were included in the final analysis.

Data collection

Data from EHRs included demographic characteristics, laboratory findings, and PSG variables. The variables collected included age, sex, nationality, body mass index (BMI), duration of diabetes, glucose-lowering agents, comorbidities (hypertension, dyslipidemia, coronary artery disease, congestive heart failure, cerebrovascular accident), and baseline clinical results (HbA1c, fasting blood glucose (FBG), blood pressure, lipid profile). OSA-related data included AHI and CPAP compliance (an average of four hours of use per night for more than 70% of the nights). Glycemic control was assessed through changes in HbA1c and FBG levels over a six-month period.

Statistical analysis

Statistical analysis was conducted using IBM SPSS version 26 (IBM Corp., Armonk, NY, USA). The normality of data was checked by the Kolmogorov-Smirnov test. Mean and standard deviation (SD) were calculated for standard data, and median (interquartile range [IQR]) was calculated for non-normal data. Frequencies and percentages were calculated for all categorical variables such as gender, age group, presence of comorbidities, OSA severity, nationality, and CPAP compliance. Comparisons were made using the Wilcoxon signed-rank test for asymmetric data and paired t-test for symmetric data.

## Results

Table 1 provides the demographic and clinical characteristics of the study participants at the start of the study. The majority were aged 41-60 (69; 61.6%), with 33 (29.5%) over 60 and 10 (8.9%) under 40. There were slightly more males (64, 57.1%). Most of the participants were Saudi (93, 83%). Eighty-four patients representing 75% of the sample were hypertensive, followed by dyslipidemia (70, 62.5%), congestive heart failure (CHF) (23, 20.5%), and coronary artery disease (CAD) (20, 17.9%). Seventy-four (66.1%) cases adhered to CPAP therapy (more than four hours/night for more than 70% of the nights). Median HbA1c was 7.2 (IQR: 6.4-8.6), and median FBG was 120 mg/dL (IQR: 105-181). Results revealed high BMI (mean 37.5 ± 6.3), severe OSA (median AHI: 40.2 events/hour), and poor sleep quality (median total sleep time (TST): five hours).

**Table 1:** Baseline Characteristics (n=112). CPAP: continuous positive airway pressure, DM: diabetes mellitus, HbA1c: hemoglobin A1c, SBP: systolic blood pressure, DBP: diastolic blood pressure, FBG: fasting blood glucose, LDL: low-density lipoprotein, HDL: high-density lipoprotein, AHI: apnea-hypopnea index, TST: total sleep time, ODI: oxygen desaturation index, SpO2: oxygen saturation in the blood, CAD: coronary artery disease, CHF: congestive heart failure, CVA: cerebrovascular accident. Data are presented as mean ± SD for normally distributed variables and median (IQR) for non-normally distributed variables, unless otherwise specified.

		Frequency
Age Mean (SD) = 55.8 (10.5)	< 40	10 (8.9%)
	41 - 60	69 (61.6%)
	> 60	33 (29.5%)
Gender	Male	64 (57.1%)
	Female	48 (42.9%)
Nationality	Saudi	93 (83%)
	Non-Saudi	19 (17%)
Co-morbidities	Hypertension	84 (75%)
	Dyslipidemia	70 (62.5%)
	CAD	20 (17.9%)
	CHF	23 (20.5%)
	CVA	9 (8%)
CPAP Compliance (> 4H/night)	Yes	74 (66.1%)
	No	38 (33.9%)
Duration of DM	Mean (SD)	13.2 ±10.2
Glucose-Lowering agents	Mean (SD)	2 ±1.1
HbA1c	Median (IQR)	7.2 (6.4 - 8.6)
FBG	Median (IQR)	120 (105 - 181)
BMI	Mean (SD)	37.5 ±6.3
SBP	Median (IQR)	132 (123 – 139)
DBP	Median (IQR)	78.5 (73.3 - 84)
Total cholesterol	Median (IQR)	155 (131 - 190)
Triglycerides	Median (IQR)	116 (94 - 153)
LDL	Median (IQR)	93 (70 - 127)
HDL	Median (IQR)	42 (37 - 49)
TST, Hours	Median (IQR)	5 (3.4 - 5.6)
AHI, events/h	Median (IQR)	40.2 (21.3 - 56.1)
4% ODI events/h	Median (IQR)	28.4 (13.3 - 48.25)
Spo2 < 90%,min	Median (IQR)	5.45 (1.2 - 17.5)
Arousal Index, events/h	Median (IQR)	55 (33 - 76.6)

Table 2 compares metabolic parameters before and after six months of CPAP therapy. CPAP adherence was defined as using the device for an average of four or more hours per night on more than 70% of nights, in accordance with standard definitions used in previous literature. HbA1c was significantly reduced from 7.15 (IQR: 6.4-8.55) to 6.9 (IQR: 6.2-7.95) (p=0.001). FBG was also decreased after six months of CPAP but was insignificant from 120 (IQR: 105-180) to 113 (IQR: 100.5-133.5) mg/dL (p=0.135). No significant changes in cholesterol, triglycerides, or blood pressure were found, and also no significant change was found in medication use (p=0.55).

**Table 2:** Effect of CPAP on Glycemic Control and Lipid Profile (n=112). CPAP: continuous positive airway pressure, HbA1c: hemoglobin A1c, SBP: systolic blood pressure, DBP: diastolic blood pressure, FBG: fasting blood glucose, LDL: low-density lipoprotein, HDL: high-density lipoprotein. P-value < 0.05 was considered as significant. Data are presented as mean ± SD for normally distributed variables and median (IQR) for non-normally distributed variables, unless otherwise specified. Statistical analysis was performed using the Wilcoxon signed-rank test for non-parametric data and paired t-test for parametric data.

	Before CPAP median (IQR)	After six months median (IQR)	p-values (Wilcoxon Test)	Test Statistics (z)
HbA1c	7.15 (6.4 - 8.55)	6.9 (6.2 - 7.95)	0.001	-3.441
FBG	120 (105 - 180)	113 (100.5 - 133.5)	0.135	-1.497
SBP	132 (124 - 141)	131.5 (123 - 139)	0.8	-0.659
DBP	77 (74 - 82.8)	78.5 (73.3 - 84)	0.95	-0.052
Total cholesterol	155 (131 - 191.5)	153 (125 - 197)	0.662	-0.437
Triglycerides	116 (94 - 154)	124 (84 - 170)	0.613	-0.506
LDL	93 (70 - 127)	99 (71 - 122)	0.786	-0.272
HDL	42 (36.5 - 49.5)	41 (35 - 48)	0.119	-1.557
Glucose-Lowering agents (Number)	1.98 (±1.1)	2 (±1.1)	0.55	-1.633

Table 3 stratifies results by CPAP compliance (four or more vs. fewer than four hours/night). In the adherent group (four or more hours), HbA1c decreased significantly from 7.55 (IQR: 6.3-9.4) to 6.25 (IQR: 6-7.8) (p<0.0001). FBG improved significantly from 149 (IQR: 105-181) to 111 (IQR: 96-133) mg/dL (p=0.049), and HDL significantly declined from 42 (IQR: 34-49) to 40 (IQR: 35-46) (p=0.049). However, SBP, DBP, total cholesterol, triglycerides, and LDL remained unchanged. In the non-adherent group (fewer than four hours), HbA1c worsened significantly from 6.8 (IQR: 6.4-8.125) to 7.2 (IQR: 6.8-8.6) (p=0.002).

**Table 3:** Effect of CPAP Adherence and Changes in HbA1c, Fasting Blood Glucose and Lipid Profile (n=112). CPAP: continuous positive airway pressure, HbA1c: hemoglobin A1c, SBP: systolic blood pressure, DBP: diastolic blood pressure, FBG: fasting blood glucose, LDL: low-density lipoprotein, HDL: high-density lipoprotein. P-value < 0.05 was considered as significant. Data are presented as mean ± SD for normally distributed variables and median (IQR) for non-normally distributed variables, unless otherwise specified. Statistical analysis was performed using the Wilcoxon signed-rank test for non-parametric data and paired t-test for parametric data.

		Before CPAP median (IQR)	After six months median (IQR)	p-values (Wilcoxon Test)	Test Statistics (z)
CPAP Compliance (≥ 4H/night) n = 74	HbA1c	7.55 (6.3 - 9.4)	6.25 (6 - 7.8)	<0.0001	-5.75
	FBG	149 (105 - 181)	111 (96 - 133)	0.049	-1.94
	SBP	132 (123 - 140.25)	133 (123.75 - 139)	0.563	-0.58
	DBP	78 (75 - 84)	79.5 (74 - 85.25)	0.25	-1.15
	Total cholesterol	170 (137 – 187)	166 (129 – 204)	0.627	-0.59
	Triglycerides	116 (94 – 157)	124 (89 – 173)	0.567	-0.70
	LDL	100 (75 - 128)	102 (77 - 130)	0.866	-0.17
	HDL	42 (34 - 49)	40 (35 - 46)	0.049	-1.97
CPAP Compliance (< 4H/night) n = 38	HbA1c	6.8 (6.4 - 8.125)	7.2 (6.8 - 8.6)	0.002	-3.07
	FBG	117 (102.3 - 188)	112 (100.5 - 164)	0.972	-0.04
	SBP	133 (124 - 142.3)	130 (119.5 - 137.3)	0.031	-2.15
	DBP	77 (73.5 - 81.25)	76 (70.5 - 81.3)	0.104	-1.63
	Total cholesterol	141 (124.5 - 200.3)	150.5 (121.3 - 188.5)	0.976	-0.03
	Triglycerides	129 (102.5 - 157.5)	125.5 (74.5 - 168)	0.841	-0.20
	LDL	100 (75 - 128)	102 (77 - 130)	0.87	-0.11
	HDL	42 (34 - 49)	40 (35 - 46)	0.049	-0.03

Table 4 analyzes CPAP effects by OSA severity (stratified by severe, moderate, and mild). In severe OSA cases, HbA1c improved significantly from 7.1 (IQR: 6.3-8.98) to 6.8 (IQR: 6.1-7.8) (p=0.002). In moderate OSA, HbA1c worsened slightly from 6.8 (IQR: 6.5-8.4) to 6.9 (IQR: 6.2-8.05) (p=0.033), while in mild OSA, no HbA1c change was noted (p=0.955).

**Table 4:** Obstructive Sleep Apnea (OSA) Severity and Changes in HbA1c and Fasting Blood Glucose (n=112). CPAP: continuous positive airway pressure, HbA1c: hemoglobin A1c, FBG: fasting blood glucose. P-value < 0.05 was considered as significant. Data are presented as mean ± SD for normally distributed variables and median (IQR) for non-normally distributed variables, unless otherwise specified. Statistical analysis was performed using the Wilcoxon signed-rank test for non-parametric data and paired t-test for parametric data.

		Before CPAP median (IQR)	After six months median (IQR)	p-values (Wilcoxon Test)	Test Statistics (z)
Severe n = 70	HbA1c	7.1 (6.3 - 8.98)	6.8 (6.1 - 7.8)	0.002	-3.09
	FBG	121.5 (105.5 - 185.8)	112.5 (94 - 138.8)	0.471	-0.72
Moderate n = 26	HbA1c	6.8 (6.5 - 8.4)	6.9 (6.2 - 8.05)	0.033	-2.13
	FBG	114 (105 - 177)	110 (97.5 - 132)	0.172	-1.37
Mild n = 16	HbA1c	7.45 (6.425 - 8.3)	7.2 (6.475 - 8.55)	0.955	-0.06
	FBG	152 (88 - 182)	112 (94.25 - 218.5)	0.499	-0.68

Table 5 compares patients with controlled (HbA1c <7%) and uncontrolled (HbA1c ≥7%) diabetes. In the uncontrolled group, HbA1c improved significantly from 8.35 (IQR: 7.7-9.95) to 7.85 (IQR: 7.23-8.9) (p=0.001), while in the controlled group, no significant changes were found.

**Table 5:** Comparison of Improvements in Glycemic Control Between Controlled vs. Uncontrolled Diabetes (n=112). CPAP: continuous positive airway pressure, DM: diabetes mellitus, HbA1c: hemoglobin A1c, SBP: systolic blood pressure, DBP: diastolic blood pressure, FBG: fasting blood glucose, LDL: low-density lipoprotein, HDL: high-density lipoprotein. P-value < 0.05 was considered as significant. Data are presented as mean ± SD for normally distributed. variables and median (IQR) for non-normally distributed variables, unless otherwise specified. Statistical analysis was performed using the Wilcoxon signed-rank test for non-parametric data and paired t-test for parametric data.

		Before CPAP median (IQR)	After six months median (IQR)	p-values (Wilcoxon Test)	Test Statistics (z)
Controlled DM n = 52	HbA1c	6.4 (6.1 - 6.7)	6.2 (6 - 6.7)	0.179	-1.24
	FBG	106 (93 - 118.5)	102 (93.5 - 112)	0.9	-0.55
	SBP	130 (122 - 137.5)	130.5 (122 - 135.75)	0.689	-1.08
	DBP	78 (75 - 82.75)	80 (74 - 85.75)	0.248	-1.08
	Total cholesterol	162 (133 - 196)	166.5 (128.3 - 202.3)	0.806	-0.47
	Triglycerides	107 (91 - 145)	113.5 (75.5 - 161.3)	0.466	-1.10
	LDL	95 (72 - 136)	105 (76.8 - 130.8)	0.925	-0.77
	HDL	44 (39 - 50)	42 (38 - 54.5)	0.789	-1.68
Uncontrolled DM n = 60	HbA1c	8.35 (7.7 - 9.95)	7.85 (7.23 - 8.9)	0.001	-4.01
	FBG	178 (144 - 213.5)	132.5 (112 - 200.5)	0.155	-1.21
	SBP	134 (125 - 143.5)	133 (125.3 - 141.8)	0.605	-0.11
	DBP	77 (74 - 82.75)	78 (72.25 - 82)	0.339	-0.98
	Total cholesterol	153 (126 - 190.75)	152 (119 - 185.5)	0.815	-1.01
	Triglycerides	127.5 (101.8 - 173.3)	143 (95.5 - 200)	0.187	-0.30
	LDL	90 (65 - 125)	91 (65.5 - 119.5)	0.774	-0.89
	HDL	41 (33.75 - 49)	41 (31.5 - 46)	0.52	-0.66

Table 6 focuses on extreme glycemic dysregulation. CPAP had dramatic effects in the highest-risk subgroup. In the HbA1c ≥9% group, a significant reduction was found in HbA1c from 10 (IQR: 9.35-11.1) to 8.6 (IQR: 7.4-9.45) (p<0.001). While in the HbA1c <9% group, no significant changes were found.

**Table 6:** Comparison of Improvements in Glycemic Control Between Controlled vs. Poorly Controlled Diabetes (n=112). CPAP: continuous positive airway pressure, DM: diabetes mellitus, HbA1c: hemoglobin A1c, SBP: systolic blood pressure, DBP: diastolic blood pressure, FBG: fasting blood glucose, LDL: low-density lipoprotein, HDL: high-density lipoprotein. P-value < 0.05 was considered as significant. Data are presented as mean ± SD for normally distributed variables and median (IQR) for non-normally distributed variables, unless otherwise specified. Statistical analysis was performed using the Wilcoxon signed-rank test for non-parametric data and paired t-test for parametric data.

		Before CPAP median (IQR)	After six months median (IQR)	p-values (Wilcoxon Test)	Test Statistics (z)
HbA1c ≥9% n = 25	HbA1c	10 (9.35 - 11.1)	8.6 (7.4 - 9.45)	<0.001	-3.91
	FBG	203 (162 - 241)	130 (110.5 - 168.25)	0.092	-1.69
	SBP	134 (124.5 - 145.5)	131 (122 - 140)	0.276	-1.09
	DBP	79 (74.5 - 86)	78 (71.5 - 84.5)	0.206	-1.26
	Total cholesterol	163 (143.3 - 186.5)	161 (120 - 214)	0.89	0.00
	Triglycerides	131.5 (107.8 - 185.3)	128 (96 - 248)	0.975	-0.03
	LDL	100 (67 - 129)	99 (58 - 122)	0.99	0.00
	HDL	40 (31.75 - 53.5)	41 (31 - 50)	0.305	-1.03
HbA1c <9% n = 87	HbA1c	6.7 (6.3 - 7.6)	6.7 (6.1 - 7.5)	0.239	-1.18
	FBG	113 (100 - 152)	110 (95 - 131.5)	0.679	-0.41
	SBP	132 (123 - 140)	132 (123 - 138)	0.869	-0.17
	DBP	77 (74 - 82)	79 (74 - 84)	0.427	-0.79
	Total cholesterol	149 (129 - 194.5)	152.5 (125.3 - 193)	0.743	-0.33
	Triglycerides	112 (91 - 147.5)	119.5 (79.3 - 166.8)	0.571	-0.57
	LDL	92 (70 - 127)	97.5 (71.25 - 123)	0.877	-0.16
	HDL	42 (37 - 49)	41.5 (37.25 - 47.5)	0.216	-1.24

Table 7 compares glycemic control (HbA1c, FBG) and lipid profiles before and after six months of CPAP therapy, stratified by BMI categories: normal-overweight (n=11) and obese (n=101). In the obese group, HbA1c significantly improved (median before: 7.4 (IQR: 6.5-8.95), after six months 6.9 (IQR: 6.2-8.25), p<0.001), and FBG was also significantly reduced (median before: 143 (IQR: 110-184.5), after: 112 (IQR: 95-143.5), p=0.045). While no significant changes were found in HbA1c or FBG (p>0.05) in the normal-overweight group, and no significant changes in total cholesterol, triglycerides, LDL, or HDL were found in either group (all p-values >0.05).

**Table 7:** Comparison of HbA1c, FBG, and Lipid Profile Between BMI Groups (n=112). CPAP: continuous positive airway pressure, DM: diabetes mellitus, HbA1c: hemoglobin A1c, SBP: systolic blood pressure, DBP: diastolic blood pressure, FBG: fasting blood glucose, LDL: low-density lipoprotein, HDL: high-density lipoprotein. P-value < 0.05 was considered as significant. Data are presented as mean ± SD for normally distributed variables and median (IQR) for non-normally distributed variables, unless otherwise specified. Statistical analysis was performed using the Wilcoxon signed-rank test for non-parametric data and paired t-test for parametric data.

		Before CPAP median (IQR)	After six months median (IQR)	p-values (Wilcoxon Test)	Test Statistics (z)
Normal – Overweight n = 11	HbA1c	6.5 (6.2 - 6.8)	6.3 (6.1 - 7.2)	0.281	-1.08
	FBG	100 (93 - 124.5)	118 (99.75 - 136.5)	0.343	-0.95
	Total cholesterol	132 (125.5 - 184)	133 (116.75 - 176.75)	0.678	-0.42
	Triglycerides	105.5 (78.25 - 159.75)	131.5 (80 - 163)	0.878	-0.15
	LDL	80.5 (69.5 - 111.75)	78.5 (62.75 - 109.5)	0.475	-0.71
	HDL	40 (35.5 - 44.75)	40 (36.75 - 43.5)	1	0.00
Obese n = 101	HbA1c	7.4 (6.5 - 8.95)	6.9 (6.2 - 8.25)	<0.001	-3.78
	FBG	143 (110 - 184.5)	112 (95 - 143.5)	0.045	-1.84
	Total cholesterol	161 (135 - 193)	159 (129 - 201)	0.777	-0.28
	Triglycerides	118 (95 - 153)	120 (84.5 - 173)	0.573	-0.56
	LDL	95 (71 - 128)	99 (73 - 123)	0.973	-0.03
	HDL	42 (36 - 50)	42 (35 - 48)	0.099	-1.65

Table 8 compares changes in glycemic and lipid parameters before and after CPAP therapy in patients with comorbidities (n=98) vs. without comorbidities (n=14). In cases with comorbidities, HbA1c significantly improved (median before: 7.2 (IQR: 6.4-9), after: 6.85 (IQR: 6.1-8.05), p<0.001). While FBG trended toward improvement (median before: 125.5 (IQR: 107-181.8), after: 112 (IQR: 96.5-140.5), p=0.065). No significant changes were found in HbA1c or FBG (p>0.05) in cases without comorbidities. There were no significant changes in total cholesterol, triglycerides, LDL, HDL, SBP, or DBP in either group (all p>0.05).

**Table 8:** Comparison of HbA1c, FBG and Lipid Profile Between With and Without Comorbidities (n=112). CPAP: continuous positive airway pressure, DM: diabetes mellitus, HbA1c: hemoglobin A1c, SBP: systolic blood pressure, DBP: diastolic blood pressure, FBG: fasting blood glucose, LDL: low-density lipoprotein, HDL: high-density lipoprotein. P-value < 0.05 was considered as significant. Data are presented as mean ± SD for normally distributed variables and median (IQR) for non-normally distributed variables, unless otherwise specified. Statistical analysis was performed using the Wilcoxon signed-rank test for non-parametric data and paired t-test for parametric data.

		Before CPAP median (IQR)	After six months median (IQR)	p-values (Wilcoxon Test)	Test Statistics (z)
With Comorbidities n=98	HbA1c	7.2 (6.4 - 9)	6.85 (6.1 - 8.05)	<0.001	-4.11
	FBG	125.5 (107 - 181.8)	112 (96.5 - 140.5)	0.065	-1.85
	SBP	132 (123.8 - 140.3)	132 (123 - 139)	0.459	-0.74
	DBP	78 (74 - 83)	78.5 (73 - 85)	0.998	-0.002
	Total cholesterol	156 (131 - 193)	152.5 (122.8 - 199.3)	0.646	-0.46
	Triglycerides	116 (92 - 153)	122 (84.25 - 169.25)	0.763	-0.30
	LDL	93 (70 - 127)	97.5 (71 - 123.5)	0.753	-0.31
	HDL	42 (35 - 50)	41 (35.5 - 47.8)	0.114	-1.58
Without Comorbidities n=14	HbA1c	7 (6.65 - 7.9)	7.2 (6.675 - 7.95)	0.238	-1.18
	FBG	106 (95.5 - 219.25)	105 (91.5 - 150.8)	0.5	-0.67
	SBP	132 (125.3 - 142.8)	132 (122 - 139.25)	0.95	-0.06
	DBP	77 (76.8 - 80)	78.5 (75.8 - 82.25)	0.91	-0.004
	Total cholesterol	144.5 (127 - 194.8)	165 (126 - 184)	0.56	-0.68
	Triglycerides	131 (108.3 - 175.5)	136 (68 - 198)	0.5	-0.67
	LDL	81 (66.8 - 124.8)	103 (71 - 118)	0.786	-0.27
	HDL	41.5 (39 - 43)	44 (34 - 54)	0.686	-0.41

## Discussion

This retrospective cohort study explored the association between CPAP therapy and glycemic control over a six-month period in patients with T2DM and OSA. The findings demonstrated that consistent use of CPAP was associated with significant improvement in glycemic parameters, particularly HbA1c levels. The study sample was predominantly middle-aged, with a slight majority of male participants, and most were of Saudi nationality. Hypertension and dyslipidemia were the most common comorbidities observed. A considerable proportion of participants adhered well to CPAP therapy, and overall, they presented with poor baseline glycemic control, high BMI, and severe OSA. The findings of this study support a meaningful association between CPAP therapy and improved glycemic control, with the magnitude of benefit varying across patient subgroups. This improvement appears more pronounced in individuals with poorer baseline glycemic control and higher levels of treatment adherence, suggesting that CPAP may confer a clinically relevant metabolic benefit when sufficient therapeutic exposure is achieved. Similar observations have been reported in prior interventional studies, in which significant reductions in HbA1c were noted mainly after sustained CPAP use and among patients with suboptimal baseline control. These observations offer important insight into the potential metabolic effects of CPAP therapy and are best understood in the context of the study’s detailed findings and the existing literature.

OSA is strongly associated with a range of cardiovascular and metabolic disorders, including hypertension, ischemic heart disease, T2DM, and dyslipidemia [17,18,20,21]. In this retrospective study, we found that the use of CPAP in patients with OSA and T2DM can have an effect on certain metabolic parameters. Based on our findings, CPAP therapy may contribute to an improvement in HbA1c levels in patients who used it for at least six months, with values decreasing from 7.15% to 6.9%. Additionally, there was a reduction in FBG from 120 mg/dL to 113 mg/dL. These results are consistent with those of a systematic review and meta-analysis, which also demonstrated that CPAP could effectively reduce HbA1c by approximately 0.24%, potentially contributing to a reduction in mortality in such patients; however, the same study reported no significant change in FBG [22]. Moreover, our study showed no significant effect of CPAP on total cholesterol, triglycerides, LDL, HDL, or blood pressure, and without statistical significance (all p > 0.05). These findings suggest that while CPAP may be beneficial for glycemic control, particularly in patients with coexisting metabolic and cardiovascular conditions, its short-term impact on blood pressure and lipid levels may be limited. This selective effect may indicate that the metabolic benefits of CPAP are more pronounced in glucose regulation than in lipid or blood pressure modulation over short-term follow-up. These outcomes are inconsistent with several previous studies that reported more favorable effects of CPAP on cardiovascular and metabolic parameters. For instance, a randomized controlled trial involving obese Chinese patients with T2DM and moderate-to-severe OSA found significant reductions in both systolic (−10 mmHg) and diastolic (−6 mmHg) blood pressure after just three months of CPAP therapy, although no significant improvements in glycemic control were observed [23]. Similarly, a separate long-term study spanning 24 months reported a modest yet significant decrease in diastolic blood pressure (−2.2 mmHg), with stronger reductions seen among individuals with higher baseline blood pressure and better CPAP adherence [24]. Additionally, another randomized controlled trial - closely aligned with our patient population in terms of disease severity and comorbidities - evaluated the impact of eight weeks of CPAP therapy in clinically stable patients with severe OSA. In that study, patients who were adherent to therapy showed significant reductions in both systolic and diastolic blood pressure (p=0.001 and p=0.006, respectively), as well as in total serum cholesterol levels (p=0.002) [25].

The differences between these findings and ours may be attributed to several factors, including treatment duration, population characteristics, and variability in CPAP adherence. Additionally, in contrast to the studies that demonstrated significant reductions in blood pressure, it is important to note that in our study, blood pressure readings were obtained from routine clinical measurements taken by nursing staff during patient visits, rather than through standardized or controlled methods. This approach may have introduced variability and reduced the accuracy of the readings, potentially obscuring any subtle changes in blood pressure attributable to CPAP therapy. Despite the absence of statistically significant improvements in blood pressure or lipid profiles in our study, the consistent reduction in HbA1c among our patient population highlights a potentially important metabolic benefit of CPAP therapy.

Regarding the impact of CPAP adherence, we evaluated changes in metabolic parameters between individuals who adhered to CPAP therapy and those who did not. We compared the baseline results before starting CPAP with the outcomes after six months of usage. The adherent group showed significantly better outcomes compared to the non-adherent group, which, in contrast, demonstrated worsening in metabolic parameters. In the adherent group, there was a significant improvement in HbA1c, which decreased from 7.55 to 6.25. There was also a favorable reduction in FBG, which dropped from 149 to 111. However, HDL showed a slight decline from 42 to 40. In the non-adherent group, HbA1c worsened, increasing from 6.8 to 7.2. These findings support the idea that better compliance with CPAP therapy is associated with improved optimization of risk factors that OSA may worsen. It is also possible that improved sleep quality and daytime alertness contributed indirectly to better glycemic control rather than reflecting a purely metabolic effect of CPAP. This is consistent with findings in the literature that suggest sleep fragmentation increases sympathetic activity, which in turn raises blood glucose level. Moreover, OSA is known to induce insulin resistance, which can cumulatively contribute to the worsening of diabetes in affected patients [26]. In contrast, a randomized controlled trial comparing two groups with T2DM and OSA over a three-month period - where the compliant group used CPAP for an average of 6.9 hours per night, and the non-compliant group averaged 3.9 hours per night - found no significant difference between the two groups in HbA1c or blood pressure levels [27].

An analysis of our data indicates that CPAP therapy had a greater impact on individuals with uncontrolled diabetes, mainly those with poor glycemic control. Patients in the uncontrolled group (HbA1c ≥7%) had a statistically significant improvement in glycemic control, with HbA1c levels decreasing from 8.35 to 7.85. On the other hand, patients in the controlled group (HbA1c <7%) showed no significant changes, suggesting that the benefits of CPAP are more evident in patients with poorer baseline glycemic control. Further analysis among our patients with HbA1c ≥9% revealed that CPAP led to a marked reduction in HbA1c, from 10 to 8.6, with a highly significant p value <0.001. Similarly, a study done by Sharma et al., which focused on patients with poorly controlled T2DM and OSA, showed that the CPAP group experienced a significant reduction in HbA1c levels in comparison to the control group [19]. Moreover, a study by Zhu et al. found that the effect of CPAP on glycemic control in well-controlled T2DM patients is not significant [28]. Taken together, these findings highlight the importance of considering baseline glycemic status when evaluating the possible benefits of CPAP therapy. Patients with poor glycemic control may experience more significant improvements, highlighting the need for targeted interventions in this group of patients.

To further explore the metabolic impact of CPAP therapy, we examined whether patients' BMI influenced their response to treatment in terms of glycemic and lipid outcomes. Results indicate that CPAP therapy has a significant effect on glycemic control in obese patients with BMI ≥30, with HbA1c decreasing from 7.4 to 6.9 (p < 0.001) and FBG from 143 to 112 (p = 0.045) after six months. However, no significant changes were observed in the normal-overweight group. This suggests that CPAP may be more beneficial for patients with higher BMI, who typically have more insulin resistance. Despite improvements in glycemic control, there were no significant changes in lipid profile in either group. Overall, these results suggest that CPAP can improve glycemic control, particularly in obese patients, but its effect on lipid profiles remains unclear. The literature remains limited in addressing how BMI influences the metabolic response to CPAP therapy. There are limited studies that stratify outcomes based on BMI categories, making it difficult to give definitive conclusions about the effect of CPAP in different BMI groups. One meta-analysis assessed the impact of CPAP on lipid profiles, with subgroup analyses based on BMI categories revealing that while CPAP treatment led to the lowering of total cholesterol levels in the high BMI group, changes in triglycerides, HDL, and LDL were not statistically significant, questioning the clinical effect of CPAP on lipid profiles [29].

Several important limitations were identified in our study. Firstly, the retrospective design inherently limits the ability to control for all potential confounding variables and introduces the possibility of missing or incomplete data. Secondly, the single-center nature of the study may restrict the generalizability of the findings, as patient characteristics, clinical practices, and adherence patterns may vary across different institutions. Moreover, the absence of a control group limits the ability to establish causality and should be considered when interpreting the findings. Additionally, although the overall sample size was sufficient, certain subgroup analyses - such as those involving patients with mild OSA, those without comorbidities, or those with poor CPAP adherence - were limited by small numbers, which may reduce statistical power and the reliability of those specific outcomes. Lastly, key lifestyle and clinical variables, including dietary habits, physical activity levels, and medication adjustments during the follow-up period, were not systematically accounted for. These uncontrolled factors may have influenced the metabolic changes observed and should be considered when interpreting the results. Addressing these limitations in future prospective, multicenter studies will be essential to validate and refine our findings.

## Conclusions

In conclusion, this retrospective study demonstrated that CPAP therapy significantly improves glycemic control in patients with obstructive sleep apnea and type 2 diabetes, as evidenced by a significant reduction in HbA1c levels following treatment. Subgroup analysis revealed that the greatest improvements were observed among patients with poor glycemic control, higher BMI, better adherence to CPAP therapy, and those with more severe OSA, highlighting the role of both disease severity and treatment compliance in optimizing outcomes. These findings underscore the importance of screening for and managing OSA in diabetic patients as part of a comprehensive approach to metabolic control. Further prospective studies with larger sample sizes and CPAP adherence tracking are warranted to confirm these results and explore the underlying mechanisms linking sleep-disordered breathing to glucose metabolism.
